# Supplementary material for: Anomalous diffusion along metal/ceramic interfaces
Source: Nat Commun. 2018 Dec 7;9:5251. doi: 10.1038/s41467-018-07724-7 (PMC6286315; doi:10.1038/s41467-018-07724-7)
Supplement: Supplementary file 1 — Supplementary Information [file 41467_2018_7724_MOESM1_ESM.pdf]

Supplementary Information (SI)

Anomalous diffusion along metal/ceramic interfaces

Kumar *et al.*

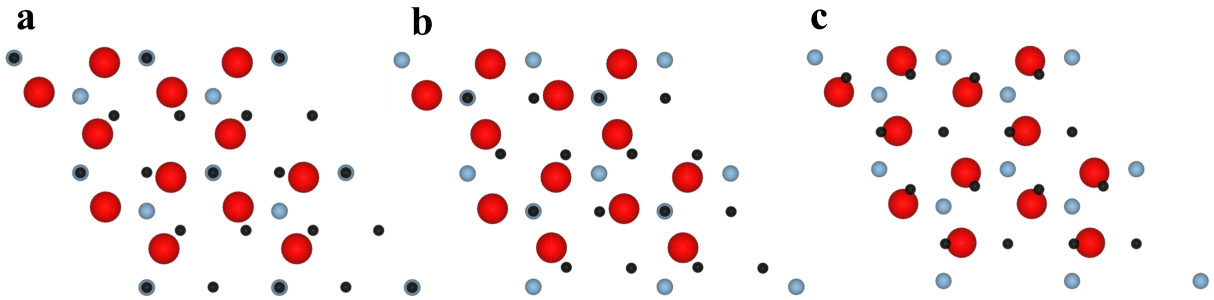

Supplementary Figure 1. Top view of the M1 Ni(111)[1$\bar{1}$0]||α-Al_2O_3(0001)[11$\bar{2}$0] (O-terminated) with the different terminating Ni {111} planes, which represent different translations of the Ni crystal parallel to the interface. Ni, Al and O are indicated by black, blue and red. The Ni is **a** A-terminated, **b** B-terminated and **c** C-terminated.

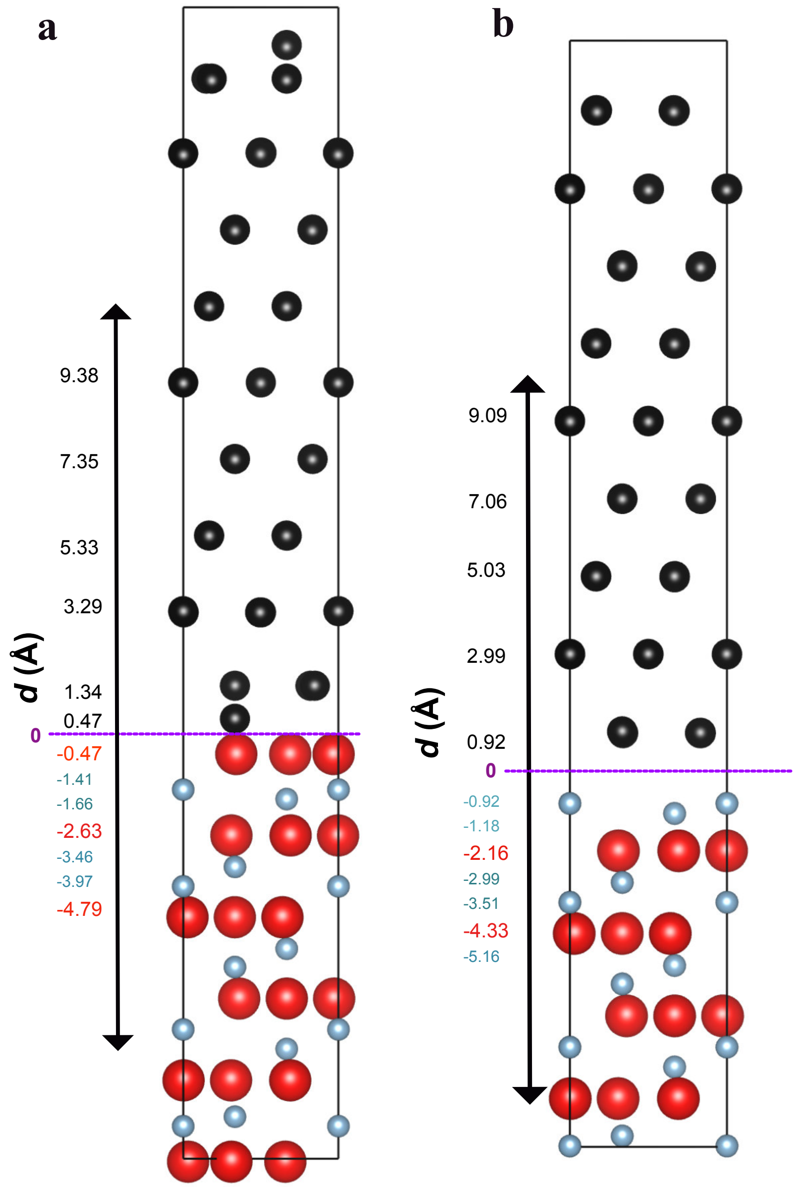

Supplementary Figure 2. Distance d of Ni, Al and O vacancies from the relaxed interface for **a** O-terminated and **b** 2Al-terminated M1 interfaces. We define d=0 as half of the interlayer distance between the terminating Ni plane (from the metal side) and Al or O plane (from the ceramic side).

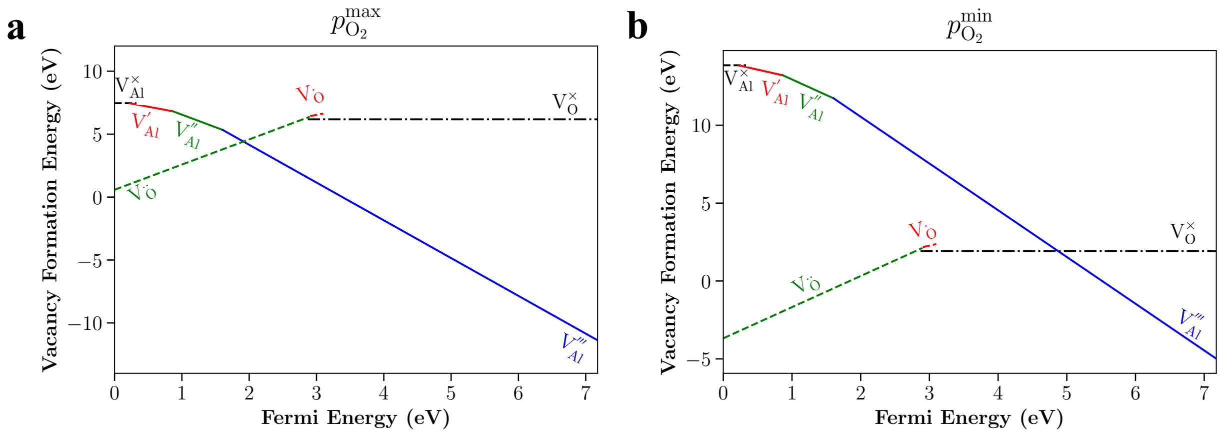

Supplementary Figure 3. Vacancy formation energies for Al and O in sapphire for different charge states according to the Kröger Vink notation at **a** $p_{O_2}^{max}$ condition and **b** $p_{O_2}^{min}$ condition.

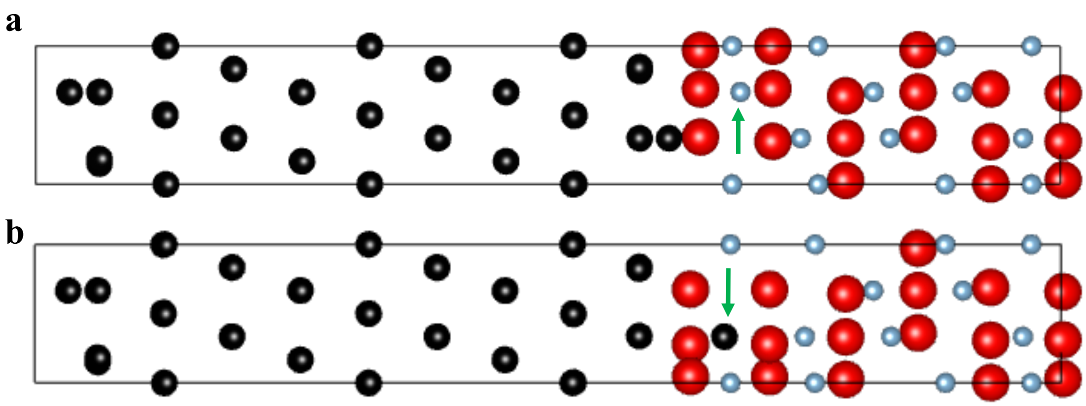

Supplementary Figure 4. Relaxation of the interface containing an Al vacancy. Ni, Al and O are indicated by black, blue and red. **a** An Al atom (indicated by the arrow) is removed to form a Al vacancy. **b** Relaxation of this interface with an Al vacancy shows that a Ni atom moves across the interface leaving behind a Ni vacancy and creating a Ni interstitial in the sapphire (indicated by the arrow).

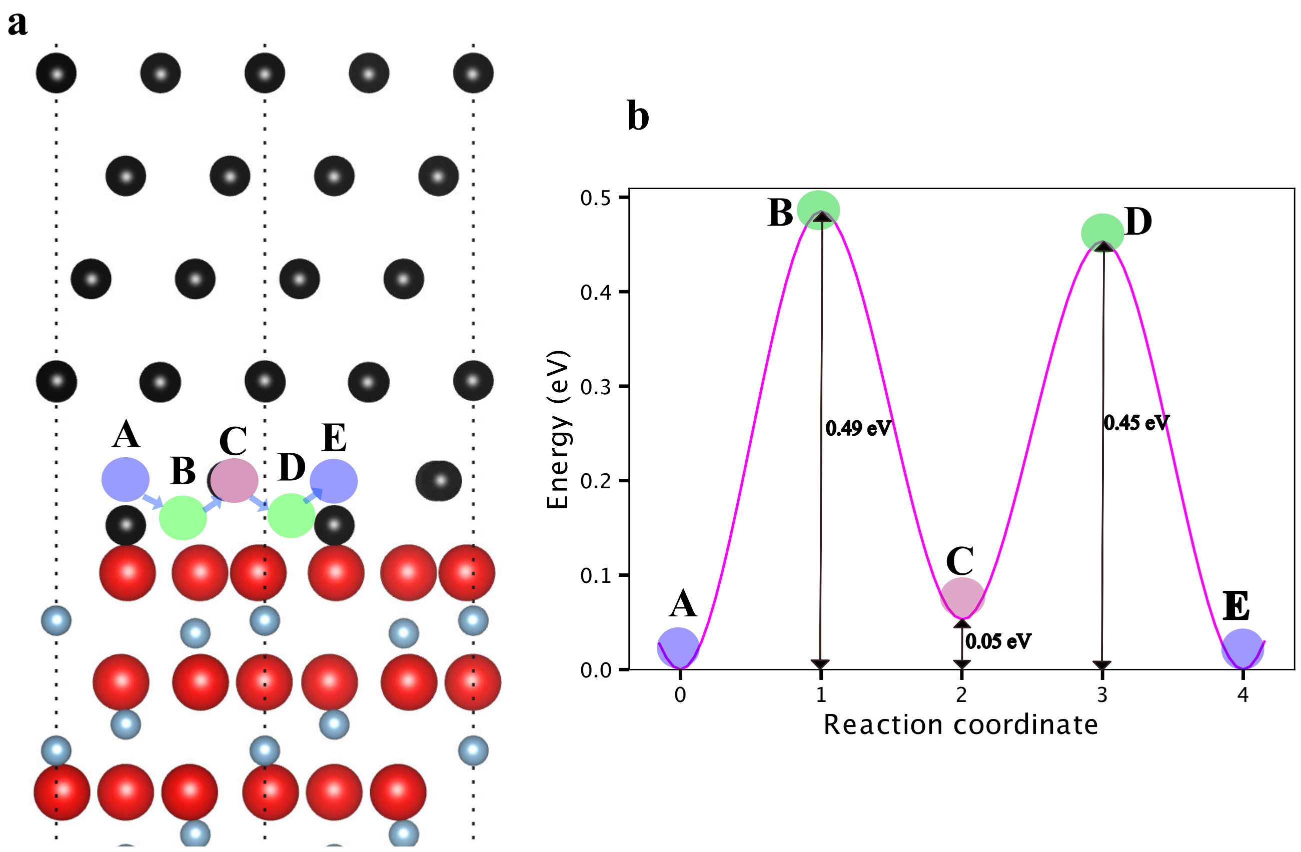

Supplementary Figure 5. DFT prediction of the Ni vacancy migration. **a** zoomed in view of the relaxed O-terminated M1 interface supercell (twice the size of the M1 supercell for vacancy formation energy calculations as indicated by the dashed lines) showing the migration path of a Ni vacancy from site A to an identical side E along the path (A→B→C→D→E). Sites A and E are identical (purple), so are B and D (green). Sites A, C and E are occupied Ni atoms in the perfect relaxed supercell. **b** calculated migration barriers of the Ni vacancy jump shown along the reaction coordinate. This path is periodic with sites A and E being the lowest energy positions.

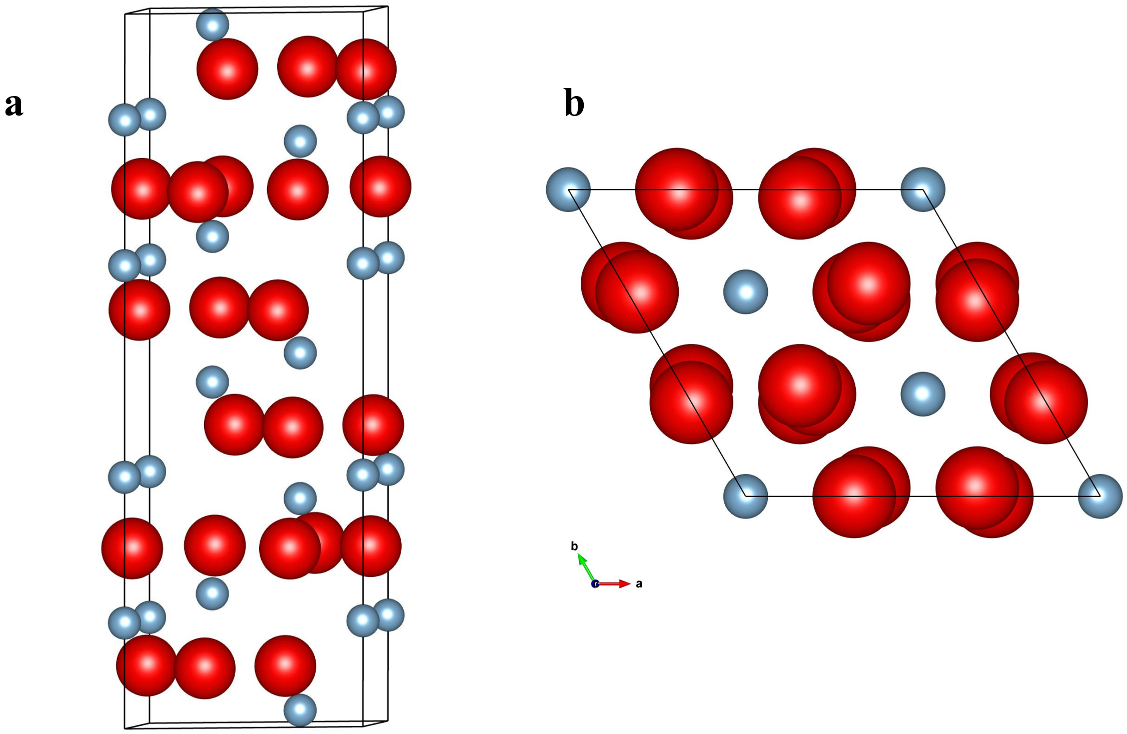

Supplementary Figure 6. Structure of α-Al_2O_3 (sapphire) that crystallizes in a trigonal structure with the space group R$\bar{3}$C, number 167. **a** side-view of the 30 atom unit cell, showing the O (red) and Al (blue) atoms. **b** top view of the same cell.

Symbol	Description
R_0	radius of the cylindrical sinking grain
R_{out}	radius of the outer grain; diffusion fluxes vanish here
$j_s(r)$	surface flux
$\mu_s(r)$	chemical potential at the surface
D_s	surface diffusion coefficient
ν_s	number of mobile atoms per unit surface area
ν_{gb}	number of mobile atoms per unit GB area
ν_i	number of mobile atoms per unit interface area
k	Boltzmann constant
T	temperature in kelvin (K)
u	velocity of the crystal lattice drift normal to the substrate
γ_{gb}	GB energy (per unit area)
j_{gb}	GB diffusion flux
D_{gb}	GB diffusion coefficient
D_i	Interface diffusion coefficient
μ_s^{gb}	chemical potential at the groove root (point A)
μ_i^{gb}	chemical potential at the intersection of the GB and the interface (point B)
h	distance between the grain boundary root (point A) and the interface
$\mu_i(r)$	chemical potential at the interface
Ω	atomic volume
$y(r,t)$	film surface profile

Supplementary Table 1. List of symbols used in the diffusion model.

Conditions	μ_O	μ_{Al}	μ_{Ni}
	(eV/atom)		
$p_{O_2}^{min}$	-11.86	-7.75	-16.07
$p_{O_2}^{max}$	-7.60	-14.13	-16.07

Supplementary Table 2. Values of the chemical potentials of O, Al and Ni for O-poor ($p_{O_2}^{min}$) and O-rich ($p_{O_2}^{max}$) conditions used to calculate the interface energies of various M1 interfaces formed by the 3 Ni terminations and the 3 sapphire terminations.

Position of the vacancy	Vacancy migration energy(eV/atom)	
	unstrained	strained(M1-epitaxy)
bulk	1.08 [1], 1.06	1.00
Coherent Twin (Σ =3)	0.99 [1]	–
LAGB (Σ =9)	0.35 [1]	–
Ni/sapphire Interface	–	0.49

Supplementary Table 3. Vacancy migration energies in bulk Ni (bulk migration calculations using PBE in [1] was carried out for a (3x3x3)a_0 cubic supercell) and two (111) Ni planes from the Ni/sapphire interface and at a coherent twin boundary and a large angle GB (LAGB) in Ni. To determine accurate migration energies in FCC Ni and along the Ni/sapphire interface, we use a supercell twice the size of that used for the Ni and M1 interface energy calculations, in b direction.

Property	PBE(GGA) (*this work)	Experiment	SCAN (this work)
a (Å)	4.81 [2], 4.81*	4.76 [3]	4.75
c (Å)	13.14 [2], 13.12*	12.99 [3]	12.96
ΔH_f (eV)	-16.71 [4], -15.5*	-17.37 [5]	-17.5
$\gamma_{(0001)}$ (J/m^2)	1.59 [6], 1.54*	2.60 [7]	1.98
E_g (eV)	5.85 [2], 5.81*	8.80 [8]	7.18

Supplementary Table 4. Properties of α-Al_2O_3 (lattice constants, formation enthalpy, surface energy for basal plane, band gap) as predicted by SCAN compared with those obtained from PBE functional and available experimental data .

Suppplementary Discussion 1: Vacancies in sapphire

The formation energy of a vacancy of charge q in a solid V_X^q can be calculated as discussed in [9]

$$E_{\mathrm{f}}^{\mathrm{V}_{\mathrm{X}}^q(\infty)} = E_{\mathrm{defect}} - E_{\mathrm{perfect}} - \Sigma n_i \mu_i + qE_F + \Delta q, \tag{1}$$

where $E_{\mathrm{f}}^{\mathrm{V}_{\mathrm{X}}^q}(\infty)$ is the bulk formation energy of the vacancy in charge state q, E_{defect} and E_{perfect} are the computed energies of the supercell with and without the defect respectively where the total volume has been fixed at the value appropriate for the perfect crystal, n_i is the number of atoms removed ($n_i < 0$) from the system to create the vacancy, μ_i is the chemical potential of the species being removed, E_F is the Fermi energy and Δq is a correction related to the finite size of the supercell [10]. For our calculations, we chose a 120 atom sapphire supercell that is a 2x2x1 replication of the 30 atom unit cell of α-$\mathrm{Al_2O_3}$ shown in Supplementary Fig. 6.

In the above equation, the chemical potential of species i is $\mu_i = \mu_{\mathrm{i}}^0 + \Delta\mu_i$. We determine the bounds on the chemical potentials for Al and O as below:

$$\mu_{\mathrm{Al}} \leq \mu_{\mathrm{Al}}^0, \quad \mu_{\mathrm{O}} \leq \mu_{\mathrm{O}}^0 \tag{2}$$

$$2\Delta\mu_{\mathrm{Al}} + 3\Delta\mu_{\mathrm{O}} = \Delta H_{f\,\mathrm{Al_2O_3}}. \tag{3}$$

Supplementary Equation (3) implies that we need know only the O or Al chemical potentials, but not both; here, we focus on the oxygen chemical potential since it is easier to manipulate experimentally (via the oxygen partial pressure).

Together, Supplementary Equations (2) and (3) can be used to set the lower and upper bounds on the oxygen partial pressures ($p_{\mathrm{O_2}}^{\mathrm{min}}$ and $p_{\mathrm{O_2}}^{\mathrm{max}}$) to ensure that sapphire is stable. For $p_{\mathrm{O_2}}^{\mathrm{min}}$, the Al chemical potential, $\mu_{\mathrm{Al}} = \mu_{\mathrm{Al}}^0$. Hence,

$$\mu_{\mathrm{O}} = \mu_{\mathrm{O}}^0 + \frac{1}{3}\Delta H_{f\,\mathrm{Al_2O_3}}. \tag{4}$$

The $p_{\mathrm{O_2}}^{\mathrm{max}}$ conditions represent the upper bound on the O chemical potential; for the Ni/$\mathrm{Al_2O_3}$ interface, we choose this as the limit above which Ni is oxidized to form NiO. Therefore, we can write,

$$\Delta\mu_{\mathrm{Ni}} + \Delta\mu_{\mathrm{O}} = \Delta H_{f\,\mathrm{NiO}}. \tag{5}$$

Combining Supplementary Equation (5) with $\mu_{\text{Ni}} = \mu^0_{\text{Ni}}$, we get

$$\mu_{\text{O}} = \mu^0_{\text{O}} + \Delta H_{f\,\text{NiO}} \tag{6}$$

and μ_{Al} can be obtained from Supplementary Equation (4). These $p^{\text{min}}_{\text{O}_2}$ and $p^{\text{max}}_{\text{O}_2}$ limits of μ_{O} are shown in Supplementary Table 1. $p^{\text{max}}_{\text{O}_2}$ favor the interface formed by O-terminated sapphire while the $p^{\text{min}}_{\text{O}_2}$ conditions lead to the interface formed by 2Al-terminated sapphire.

The Fermi energy plays an important role when the vacancy is charged — see Supplementary Equation (1). For the range of point defect energies in sapphire, the Fermi energy may vary from the valence band maximum (VBM) to the conduction band minimum (CBM) or

$$0 \leq E_F \leq 7.18\,\text{eV}. \tag{7}$$

By varying the Fermi energy (electron chemical potential), we can compare the formation energy of defects in different charge states and also locate the points of transitions from one charge state to another.

We calculate the Al vacancy (charge states 0, -1, -2 and -3, represented in Kröger-Vink notation as $\text{V}^{\times}_{\text{Al}}$, $\text{V}^{'}_{\text{Al}}$, $\text{V}^{''}_{\text{Al}}$, and $\text{V}^{'''}_{\text{Al}}$) and O vacancy (charge states 0, +1 and +2, i.e., $\text{V}^{\times}_{\text{O}}$, $\text{V}^{\cdot}_{\text{O}}$, and $\text{V}^{\cdot\cdot}_{\text{O}}$) formation energies in the 120 atom sapphire supercell. As previously discussed, the chemical potential of O can be varied between the two extremes, $p^{\text{min}}_{\text{O}_2}$ (O-poor) and $p^{\text{max}}_{\text{O}_2}$ (O-rich). Supplementary Fig. 3 shows the formation energy of vacancies in pure sapphire under these conditions. The stable point vacancies are $\text{V}^{\times}_{\text{O}}$ and $\text{V}^{'''}_{\text{Al}}$ near the mid-point of the band gap and $\text{V}^{\cdot\cdot}_{\text{O}}$ and $\text{V}^{\times}_{\text{Al}}$ near the valence band edge. $\text{V}^{\cdot}_{\text{O}}$ is never stable - consistent with earlier calculations [11]. Since we focus on vacancies in sapphire near the Ni/sapphire interface, we only consider neutral vacancies (as discussed in the manuscript).

Suppplementary Discussion 2: Interface energy

The α-Al_2O_3 (0001) free surface has received extensive attention in the literature and the Al-terminated stoichiometric free surface was determined to be most stable over the entire range of oxygen chemical potentials for which α-Al_2O_3 is stable. However, at the Ni(111)/α-Al_2O_3(0001) interface, the Al_2O_3 may have different terminations depending on the oxygen chemical potential. The Ni/sapphire (0001) interface energy γ_i for different interface terminations may be used to determine the stable interface structures at different chemical potentials, as discussed in the main text. The interface energy can be calculated as,

$$\gamma_{\mathrm{i}} = \frac{(G_0 - N_O\mu_{\mathrm{O}} - N_{\mathrm{Al}}\mu_{\mathrm{Al}} - N_{\mathrm{Ni}}\mu_{\mathrm{Ni}})}{A}, \tag{8}$$

where A is the interface area, N_{O}, N_{Al}, N_{Ni} are the number of O, Al and Ni atoms in the system and, μ_{O}, μ_{Al} and μ_{Ni} are the chemical potentials of O, Al and Ni. G_0 is the total energy of the interface from the DFT calculations. Using the constraint 2 μ_{Al}+3$\mu_{\mathrm{O}} = \mu_{\mathrm{Al_2O_3}}$, we can rewrite Supplementary Equation (8) as Supplementary Equation (9).

$$\gamma_{\mathrm{i}} = \frac{(G_0 - \frac{1}{2}N_{\mathrm{Al}}\mu^0_{\mathrm{Al_2O_3}} - [N_{\mathrm{O}} - \frac{3}{2}N_{\mathrm{Al}}]\mu_{\mathrm{O}} - N_{\mathrm{Ni}}\mu_{\mathrm{Ni}})}{A} \tag{9}$$

Supplementary Equations (2), (3) and (5) from Supplementary Discussion 1 define the range of oxygen chemical potentials (μ_{O}) of interest. The interface energies for all the interfaces formed by the three sapphire(0001) and the three Ni terminations are shown in Fig. 3b of the main text. Both the 2Al-terminated and O-terminated sapphire interfaces have a large stability region (see main text).

Supplementary Methods

Supplementary Method 1. Diffusion model

Our model describes the evolution of the surface topography of a thin metal film deposited on a ceramic substrate via simultaneous surface, grain boundary (GB), and interface self-diffusion of the film material (diffusion within the metal film grains and in the substrate are assumed to be zero). The analyzed system (Fig. 2.a, manuscript) consists of a cylindrical metal grain of constant radius R_0 embedded in a continuous metal film. We explicitly assume that at the outer (circular) edge of the continuous metal film (i.e., at R_{out}) both surface and interface diffusion fluxes vanish. This radius may be thought of as half the distance between neighboring sinking grains. The substrate is assumed to be immobile during annealing. Supplementary Table 1 introduces all of the symbols used in the model.

Surface diffusion

The driving force for surface diffusion is the curvature of the surface (i.e., capillarity; the Gibbs-Thomson effect). Therefore, the surface flux and the chemical potential at the surface are given by:

$$j_s(r) = -\frac{D_s \nu_s}{kT} \frac{\partial \mu_s}{\partial r} \tag{10}$$

$$\mu_s(r) = \Omega \gamma_s \frac{\partial}{r \partial r} \left(r \frac{\partial y}{\partial r} \right). \tag{11}$$

Within the small-slope approximation, the evolution of the film surface profile $y(r,t)$ with respect to the immobile substrate is described by:

$$\frac{\partial y}{\partial t} = -\Omega \frac{\partial}{r \partial r} (r j_s) + u, \tag{12}$$

where u is the lattice drift due to the accretion of atoms at the metal/ceramic interface.

We employ the following boundary conditions for Supplementary Equations (10) and (12):

$$\left. \frac{\partial y}{\partial r} \right|_{r=0} = \left. \frac{\partial y}{\partial r} \right|_{r=R_{out}} = 0; \qquad j_s|_{r=0} = j_s|_{r=R_{out}} = 0 \tag{13}$$

$$\left.\frac{\partial y}{\partial r}\right|_{r=R_0+} - \left.\frac{\partial y}{\partial r}\right|_{r=R_0-} = \gamma_{gb}/\gamma_s; \qquad j_s|_{r=R_0+} - j_s|_{r=R_0-} = -j_{gb}. \tag{14}$$

The boundary conditions in Supplementary Equation (13) follow from the symmetry of the system and the definition of R_{out}. The boundary conditions in Supplementary Equation (14) results from the equilibrium and mass balance conditions at the root of GB groove (point A).

Grain boundary diffusion

We assume that the material diffusing along the grain boundary does not accumulate there (otherwise, large bi-axial stresses would develop in the film; these would shutdown any mass accumulation along the GB [12]). The atoms diffusing along the grain boundary arrive at the film-substrate interface and may diffuse along the interface. Any atom accumulation along the interface will cause an upward drift of the crystal lattice of the film and concomitant change of the surface profile (note that, unlike at the GB, atom accumulation at the interface may occur because the surface is traction free). The rate of this upward drift is obtained from the mass conservation condition:

$$u = j_{gb}\,\frac{2\pi R_0}{\pi R_{out}^2}, \tag{15}$$

where j_{gb} is the constant grain boundary flux

$$j_{gb} = \frac{D_{gb}\nu_{gb}}{kT}\,\frac{\mu_s^{gb} - \mu_i^{gb}}{h}. \tag{16}$$

It should be noted that the continuity of chemical potential implies that it is the same on all sides of the groove root, which means $\mu_s^{gb} \equiv \mu_s(R_0^+) = \mu_s(R_0^-)$.

Interface diffusion

The diffusion flux along the metal/ceramic interface, j_i, is given by

$$j_i(r) = -\frac{D_i\nu_i}{kT}\,\frac{\partial \mu_i}{\partial r}. \tag{17}$$

The material accumulation at the interface should be uniform (otherwise, large bending stresses would develop in the film, leading to a large strain energy); this implies that the interface diffusion flux divergence is constant:

$$\frac{1}{r}\frac{\partial(rj_i)}{\partial r} = const. \tag{18}$$

Employing arguments similar to those used in formulation of boundary conditions in Supplementary Equations (13)-(14) yields the following set of boundary conditions for diffusion along the interface shown in Supplementary Equation (18):

$$j_i(R_{out}) = j_i(0) = 0; \qquad\qquad j_i(R_0+) - j_i(R_0-) = j_{gb}. \tag{19}$$

Combining Supplementary Equations (18) and (19) yields the following expression for the interface diffusion flux:

$$j_i(r) = -j_{gb}\,\frac{R_0}{R_{out}^2}\begin{cases} r & 0 < r < R_0 \\ r - \frac{R_{out}^2}{r} & R_0 < r < R_{out}. \end{cases} \tag{20}$$

Combining Supplementary Equations (17) and (20) and the condition of chemical potential continuity $\left(\mu_i^{gb} \equiv \mu_i(R_0+) = \mu_i(R_0-)\right)$ results in the following expression for the interface chemical potential:

$$\frac{D_i\nu_i}{kT}\,\mu_i(r) = \frac{D_i\nu_i}{kT}\,\mu_i^{gb} - j_{gb}\,\frac{R_0}{R_{out}^2}\begin{cases} 0.5(R_0^2 - r^2) & 0 < r < R_0 \\ 0.5(R_0^2 - r^2) + R_{out}^2\ln(r/R_0) & R_0 < r < R_{out}. \end{cases} \tag{21}$$

Connection between grain boundary and interface diffusion

The average value of the interface chemical potential can be determined using a thought experiment in which an infinitesimally thin disc of film material is inserted at the interface. The total energy of the system would then increase due to the formation of a new grain boundary segment around the cylindrical grain. If this increase is exactly compensated by

the energy change due to the influx of interface atoms [13, 14], we find:

$$\int_0^{R_{out}} 2\pi r \mu_i(r) dr = 2\pi R_0 \gamma_{gb} \Omega. \tag{22}$$

Combining Supplementary Equations (16),(21) and (22) gives an expression for the GB diffusion flux as a function of the material parameters ($D_{gb}, \nu_{gb}, D_i, \nu_i, \gamma_{gb}$), the geometry of the system (h, R_0, R_{out}), and only one chemical potential (linking the surface and interface diffusion, μ_s^{gb}):

$$j_{gb} = \frac{D_{gb}\nu_{gb} D_i \nu_i \left[\mu_s^{gb} - 2R_0\gamma_{gb}\Omega/R_{out}^2\right]}{kT\left[hD_i\nu_i + R_0 D_{gb}\nu_{gb}\ f(\frac{R_0}{R_{out}})\right]}, \tag{23}$$

where $f(x) = x^2 - 3/4 - \ln x$. Combining Supplementary Equations (10), (11), (15) and (23) yields a second order differential equation (12), the solution of which is $y(r,t)$.

In Fig. 2b of the manuscript, the calculated $y(r,t)$ profiles for three different values of interface diffusivities are plotted at a time corresponding to when the GB groove root arrives at the interface. This corresponds to hole nucleation and the onset of solid state dewetting. The values of the grain boundary and surface self-diffusivities (defined as the product of the diffusion coefficient, the number of mobile atoms per unit area, and the atomic volume) employed in simulations were 1.2×10^{-21}m^3/s and 10^{-21}m^3/s respectively [15, 16]. The surface and grain boundary energies of Ni were taken to be 2.1 J/m^2 and 1 J/m^2, respectively [17, 18]. The inner and outer grain diameters were set to $R_0 = 0.25$ μm and $R_{out} = 3$ μm, respectively. The three profiles in Fig. 2b of the manuscript correspond to three value of the interface diffusivity, $D_i = 0$, $D_i = D_{gb}$ and $D_i = 5D_{gb}$, at the times that the GB groove hits the substrate, i.e., 814 s, 480 s, and 232 s, respectively. One can see that increasing the interface diffusivity shortens the time needed for nucleation of a hole. Also, the amplitude of the hole rim elevation decreases with increasing interface diffusivity, creating an illusion of the ‘mass deficit’. It should also be noted that due to the axisymmetric geometry of the problem the upward drift of the film is very small and hardly discernible in this figure.

In our experiments, we determined the volume of Ni accumulated in the ridge surrounding the hole, and the volume of Ni rejected by the hole, with respect to the average height of the unperturbed thin film surface far from the hole. To mimic this experimental procedure, we performed a similar mass balance analysis employing the simulated profiles $y(r,t)$, and

using $y\,(r = R_{out}, t)$ as a reference "zero" point — resulting dependence of the volume imbalance ΔV on annealing time shown in Fig. 2c (main text). The apparent mass imbalance increases with increasing interface diffusivity. The simulations reproduce the experimentally determined mass imbalance ($5 \times 10^{-3} \mu\text{m}^3$ of Ni after 10 min annealing) for $D_i \approx D_{gb}$. Thus, the self-diffusion coefficient of Ni along the Ni-sapphire interface at 700°C is of the same order of magnitude as the self-diffusion coefficient along a random large angle grain boundary in Ni.

Supplementary Method 2. Vacancy formation energy from a generalized bond breaking model

We develop a simple, heuristic, bond breaking model to describe the vacancy formation energy at metal/ceramic interfaces. To form a vacancy within a metal requires breaking N_b metal-metal bonds, $E_{\mathrm{f}}^{\mathrm{V}_{\mathrm{m}}^{\times}}(\infty) = N_b e_{\mathrm{mm}}$, where e_{mm} is a metal-metal (mm) bond energy. (NB: the effective number of bonds broken depends on the nature of bonding; for covalent bonds it proportional to the number of nearest neighbors z, whilst in metals, where bonding is more delocalized, it is more complicated, e.g., see [19].) The metal surface energy γ_{m} in such a model is the energy to break N_s mm bonds per unit area $\gamma_{\mathrm{m}} = N_s e_{\mathrm{mm}} \rho$, where ρ is the planar density of atoms on the surface. Hence, $E_{\mathrm{f}}^{\mathrm{V}_{\mathrm{m}}^{\times}}(\infty) \approx (N_b/N_s)\gamma_{\mathrm{m}}/\rho$. The metal-ceramic (mc) interface energy is $\gamma_{\mathrm{i}} = \gamma_{\mathrm{m}} + \gamma_{\mathrm{c}} - N_i e_{\mathrm{mc}} \rho$, where γ_{c} is the surface energy of the ceramic, N_i is the number of mc bonds, and e_{mc} is the metal-ceramic bond energy. The metal-ceramic work of adhesion is $W_{\mathrm{ad}} = \gamma_{\mathrm{m}} + \gamma_{\mathrm{c}} - \gamma_{\mathrm{i}} = N_i e_{\mathrm{mc}} \rho$. Hence the metal vacancy formation energy on the metal side of the mc interface implies breaking N_i mc and $(N_b - N_s)$ mm bonds: $E_{\mathrm{f}}^{\mathrm{V}_{\mathrm{m}}^{\times}}(0) = N_i e_{\mathrm{mc}} + (N_b - N_s)e_{\mathrm{mm}} = [(N_b - N_s)\gamma_{\mathrm{m}} + N_s W_{\mathrm{ad}}]/N_s \rho$. The ratio of the metal vacancy formation energy at the metal/ceramic interface to that in the bulk metal can then be expressed as $E_{\mathrm{f}}^{\mathrm{V}_{\mathrm{m}}^{\times}}(0)/E_{\mathrm{f}}^{\mathrm{V}_{\mathrm{m}}^{\times}}(\infty) = (N_b - N_s)/N_b + (N_s/N_b)W_{\mathrm{ad}}/\gamma_{\mathrm{m}}$. Of course, a simplistic bond model is only heuristic; we therefore, only retain the basic functional form: $E_{\mathrm{f}}^{\mathrm{V}_{\mathrm{m}}^{\times}}(0)/E_{\mathrm{f}}^{\mathrm{V}_{\mathrm{m}}^{\times}}(\infty) = A + B\,(W_{\mathrm{ad}}/\gamma_{\mathrm{m}})$.

To find the two constants A and B, we augmented our Ni/Al_2O_3 calculations with similar calculations for Cu/Al_2O_3 in the same orientation and obtained W_{ad} from experiment [20] and γ_{m} from DFT [21]. This yields $A = 0.03$ and $B = 1.09$. Given the accuracy of these calculations, we approximate this as $A = 0$ and $B = 1$; which yields the simple result presented as Eq. (1) in the manuscript:

$$\frac{E_{\mathrm{f}}^{\mathrm{V}_{\mathrm{m}}^{\times}}(0)}{E_{\mathrm{f}}^{\mathrm{V}_{\mathrm{m}}^{\times}}(\infty)} = \frac{W_{\mathrm{ad}}}{\gamma_{\mathrm{m}}}. \tag{24}$$

Supplementary References

---

[1] Alexandrov, V., Sushko, M. L., Schreiber, D. K., Bruemmer, S. M. & Rosso, K. M. Ab initio modeling of bulk and intragranular diffusion in ni alloys. *The Journal of Physical Chemistry Letters* **6**, 1618–1623 (2015).

[2] Sokol, A. A., Walsh, A. & Catlow, C. R. A. Oxygen interstitial structures in close-packed metal oxides. *Chemical Physics Letters* **492**, 44–48 (2010).

[3] Shvyd'ko, Y. V. *et al.* Measuring wavelengths and lattice constants with the Mössbauer wavelength standard. *Journal of Synchrotron Radiation* **9**, 17–23 (2002).

[4] Matsunaga, K., Tanaka, T., Yamamoto, T. & Ikuhara, Y. First-principles calculations of intrinsic defects in Al_2O_3. *Physical Review B* **68**, 085110 (2003).

[5] Lide, D. R. CRC handbook of chemistry and physics: A ready-reference book of chemical and physical data (2004).

[6] Siegel, D. J., Hector Jr, L. G. & Adams, J. B. Adhesion, atomic structure, and bonding at the Al (111)/α-Al_2O_3 (0001) interface: A first principles study. *Physical Review B* **65**, 085415 (2002).

[7] McHale, J., Auroux, A., Perrotta, A. & Navrotsky, A. Surface energies and thermodynamic phase stability in nanocrystalline aluminas. *Science* **277**, 788–791 (1997).

[8] French, R. H. Electronic band structure of Al_2O_3, with comparison to AlON and AIN. *Journal of the American Ceramic Society* **73**, 477–489 (1990).

[9] Freysoldt, C. *et al.* First-principles calculations for point defects in solids. *Reviews of Modern Physics* **86**, 253 (2014).

[10] Makov, G. & Payne, M. Periodic boundary conditions in ab initio calculations. *Physical Review B* **51**, 4014 (1995).

[11] Lee, D., DuBois, J. L. & Lordi, V. Identification of the local sources of paramagnetic noise in superconducting qubit devices fabricated on α-Al_2O_3 substrates using density-functional calculations. *Physical Review Letters* **112**, 017001 (2014).

[12] Klinger, L. & Rabkin, E. Theory of the kirkendall effect during grain boundary interdiffusion. *Acta Materialia* **59**, 1389–1399 (2011).

[13] Carter, W. C., Roosen, A. R., Cahn, J. W. & Taylor, J. E. Shape evolution by surface diffusion and surface attachment limited kinetics on completely faceted surfaces. *Acta metallurgica et materialia* **43**, 4309–4323 (1995).

[14] Klinger, L. & Rabkin, E. Effects of surface anisotropy on grain boundary grooving. *Interface science* **9**, 55–63 (2001).

[15] Divinski, S. V., Reglitz, G. & Wilde, G. Grain boundary self-diffusion in polycrystalline nickel of different purity levels. *Acta Materialia* **58**, 386–395 (2010).

[16] Blakely, J. & Mykura, H. Surface self diffusion measurements on nickel by the mass transfer method. *Acta Metallurgica* **9**, 23–31 (1961).

[17] Meltzman, H., Chatain, D., Avizemer, D., Besmann, T. M. & Kaplan, W. D. The equilibrium crystal shape of nickel. *Acta Materialia* **59**, 3473–3483 (2011).

[18] Prokoshkina, D., Esin, V., Wilde, G. & Divinski, S. Grain boundary width, energy and self-diffusion in nickel: effect of material purity. *Acta Materialia* **61**, 5188–5197 (2013).

[19] Finnis, M. & Sinclair, J. A simple empirical n-body potential for transition metals. *Philosophical Magazine A* **50**, 45–55 (1984).

[20] Chatain, D., Rivollet, I. & Eustathopoulos, N. Adhésion thermodynamique dans les systèmes non-réactifs métal liquide-alumine. *Journal de Chimie Physique* **83**, 561–567 (1986).

[21] Tran, R. *et al.* Surface energies of elemental crystals. *Scientific Data* **3**, 160080 (2016).
